# Activated niacin receptor HCA2 inhibits chemoattractant-mediated macrophage migration via Gβγ/PKC/ERK1/2 pathway and heterologous receptor desensitization

**DOI:** 10.1038/srep42279

**Published:** 2017-02-10

**Authors:** Ying Shi, Xiangru Lai, Lingyan Ye, Keqiang Chen, Zheng Cao, Wanghua Gong, Lili Jin, Chunyan Wang, Mingyong Liu, Yuan Liao, Ji Ming Wang, Naiming Zhou

**Affiliations:** 1 College of Life Sciences, Zhejiang University, Yu Hang Tang Load 388, Hangzhou, PR China; 2 Cancer and Inflammation Program, Center for Cancer Research, National Cancer Institute-Frederick, NIH, Frederick, MD 21702, USA; 3 Xuzhou Yes Biotech Laboratories Ltd. Xuzhou, Jiangsu, PR China; 4 Department of Spine Surgery, Daping Hospital, Third Military Medical University, Chongqing, PR China

## Abstract

The niacin receptor HCA2 is implicated in controlling inflammatory host responses with yet poorly understood mechanistic basis. We previously reported that HCA2 in A431 epithelial cells transduced Gβγ-protein kinase C- and Gβγ-metalloproteinase/EGFR-dependent MAPK/ERK signaling cascades. Here, we investigated the role of HCA2 in macrophage-mediated inflammation and the underlying mechanisms. We found that proinflammatory stimulants LPS, IL-6 and IL-1β up-regulated the expression of HCA2 on macrophages. Niacin significantly inhibited macrophage chemotaxis in response to chemoattractants fMLF and CCL2 by disrupting polarized distribution of F-actin and Gβ protein. Niacin showed a selected additive effect on chemoattractant-induced activation of ERK1/2, JNK and PI3K pathways, but only the MEK inhibitor UO126 reduced niacin-mediated inhibition of macrophage chemotaxis, while activation of ERK1/2 by EGF alone did not inhibit fMLF-mediated migration of HEK293T cells co-expressing HCA2 and fMLF receptor FPR1. In addition, niacin induced heterologous desensitization and internalization of FPR1. Furthermore, niacin rescued mice from septic shock by diminishing inflammatory symptoms and the effect was abrogated in HCA2^−/−^ mice. These results suggest that Gβγ/PKC-dependent ERK1/2 activation and heterologous desensitization of chemoattractant receptors are involved in the inhibition of chemoattractant-induced migration of macrophages by niacin. Thus, HCA2 plays a critical role in host protection against pro-inflammatory insults.

Nicotinic acid (niacin; vitamin B3) has been used to treat dyslipidemia for more than half a century^1^,^2^, by reducing atherogenic lipoproteins LDL-c, VLDL-c, triglycerides and lipoprotein-A as well as raising plasma HDL-c^3^,^4^. Two recent meta-analyses have shown benefits of niacin in reducing cardiovascular morbidity and mortality in patients with coronary heart disease^5^,^6^,^7^,^8^,^9^,^10^. However, the mechanisms of niacin’s therapeutic effect were not well understood until the discovery of hydroxy-carboxylic acid receptor 2 (HCA2) (also known as GPR109A or HM74a in human and PUMA-G in mice) as a receptor with high affinity for niacin^11^,^12^,^13^,^14^. Subsequently, the ketone body β-hydroxybutyrate was also identified as a physiological ligand for HCA2^15^. In adipocytes, HCA2 acts via Gi proteins to decrease intracellular cAMP production in response to niacin, leading to decreased activity of hormone-sensitive lipase and reduced triglyceride hydrolysis to free fatty acids^16^.

The notion that the beneficial cardiovascular effects of niacin are linked to its antilipolytic activity via HCA2 was recently challenged by the observation that niacin exerts its activity on plasma cholesterol and triglyceride levels independently of HCA2-mediated anti-lipolytic activity and by findings that synthetic HCA2 agonists do not alter HDL-cholesterol levels in human although leading to inhibition of lipolysis^17^. However, these results did not exclude the possibility that niacin provides cardiovascular benefits in a lipid-independent manner through HCA2. In addition, Lukasova *et al*. have recently demonstrated that niacin reduces the progression of atherosclerosis in mice via HCA2 expressed by bone marrow-derived immune cells without affecting the plasma lipid profile^18^. Furthermore, HCA2 is highly expressed by immune cells including neutrophils, macrophages, and Langerhans cells as well as keratinocytes^19^,^20^,^21^. Under inflammatory conditions, elevated secretion of proinflammatory cytokines and chemokines from adipose tissue and immune cells significantly contributes to inflammation in atherosclerosis^22^,^23^. Niacin has been shown to suppress TNF-α-induced release of proinflammatory chemokines thus reducing chemokine MCP-1 (CCL2)-induced recruitment of macrophages into the atherosclerotic lesions^18^,^24^,^25^,^26^. Interestingly, recent *in vitro* and *in vivo* studies have revealed anti-inflammatory roles of niacin in acute ischemic stroke, arthritis, chronic renal failure and sepsis^27^,^28^,^29^,^30^,^31^,^32^, suggesting that niacin not only inhibits vascular inflammation, but also has therapeutic potential in other systemic inflammatory and metabolic diseases.

Macrophages have been shown to play a central role in atherosclerosis and are associated with HCA2-mediated effects independent of the antilipolytic activity of HCA2 agonists^18^,^24^,^25^,^26^,^33^,^34^. We therefore further characterized the effects of niacin on macrophages and explored the downstream signaling pathways of HCA2 involved in anti-inflammatory effects. In this study, we demonstrate that HCA2 on macrophages is significantly up-regulated by LPS, IL-6 or IL-1β and mediates an inhibitory activity of niacin on chemoatractant-induced macrophage migration. The effect of niacin on macrophage chemotaxis is independent of the polymerization of F-actin, but is due to the suppression of F-actin and Gβ protein polarization. ERK1/2 activation and heterologous desensitization of chemoattractant receptors are involved in the inhibition of chemoattractant-induced migration of macrophages by activated HCA2. *In vivo*, niacin protects the host from septic shock through HCA2. Thus, our study revealed a novel molecular mechanism of HCA2 in controlling inflammatory responses.

## Results

### HCA2 expression is enhanced in macrophages by proinflammatory stimulants

**Figure 1:**
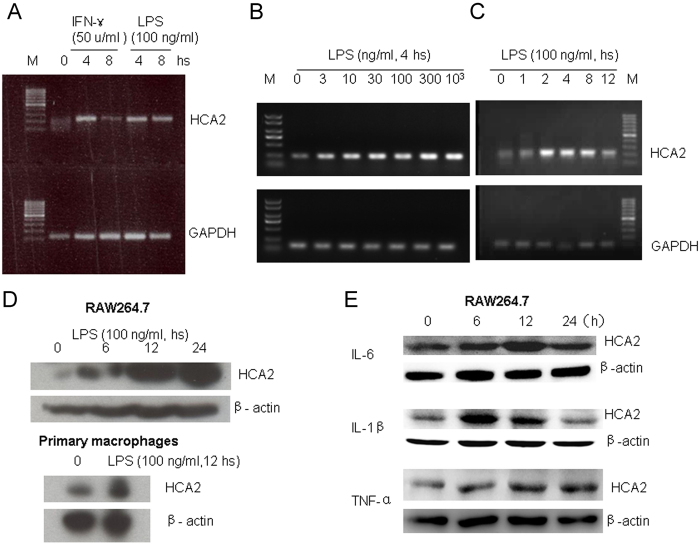
The expression of HCA2 by RAW264.7 macrophages measured by RT-PCR (**A–C**) and Western blot (**D–E**). (**A**) RAW264.7 cells stimulated by IFN-γ (50 U/ml) and LPS (100 ng/ml) for 4 or 8 hs. (**B**) RAW264.7 cells stimulated by different concentrations of LPS for 4 hs. (**C**) RAW264.7 cells stimulated by 100 ng/ml LPS for different times. (**D**). RAW264.7 cells treated with 100 ng/ml LPS for 0, 6, 12 or 24 hs and primary macrophages isolated from mice treated with or without 100 ng/ml LPS for 12 hs. (**E**) RAW264.7 cells treated with 100 ng/ml IL-6, 10 ng/ml IL-1β and 10 ng/ml TNF-α separately for 0, 6, 12 or 24 hs.

HCA2 expression was reported to be upregulated in murine macrophage cell lines ANA-1 and RAW264.7 by stimulation of IFN-γ in combination with TNF-α or LPS alone [20]. We therefore examined the capacity of IL-6 and IL-1β to induce HCA2 in mouse macrophages. Figure 1 shows that unstimulated mouse RAW264.7 cells expressed low levels of HCA2 mRNA (Fig. 1A and B) and protein (Fig. 1C and D). The expression was significantly enhanced following stimulation by LPS or IFN-γ alone. Consistent with the findings in RAW264.7 cell line, LPS also markedly increased HCA2 in primary mouse macrophages (Fig. 1D). Furthermore, HCA2 protein significantly increased in RAW264.7 cell line by IL-6 or IL-1β stimulation, but had no significant change with TNF-α alone (Fig. 1E). Thus, HCA2 expression in macrophages is enhanced by a range of proinflammatory stimulants including LPS, IL-6 and IL-1β.

### The HCA2 agonist niacin inhibits macrophage chemotaxis in response to fMLF and CCL2 by disruption of F-actin and Gβ protein polarization

**Figure 2:**
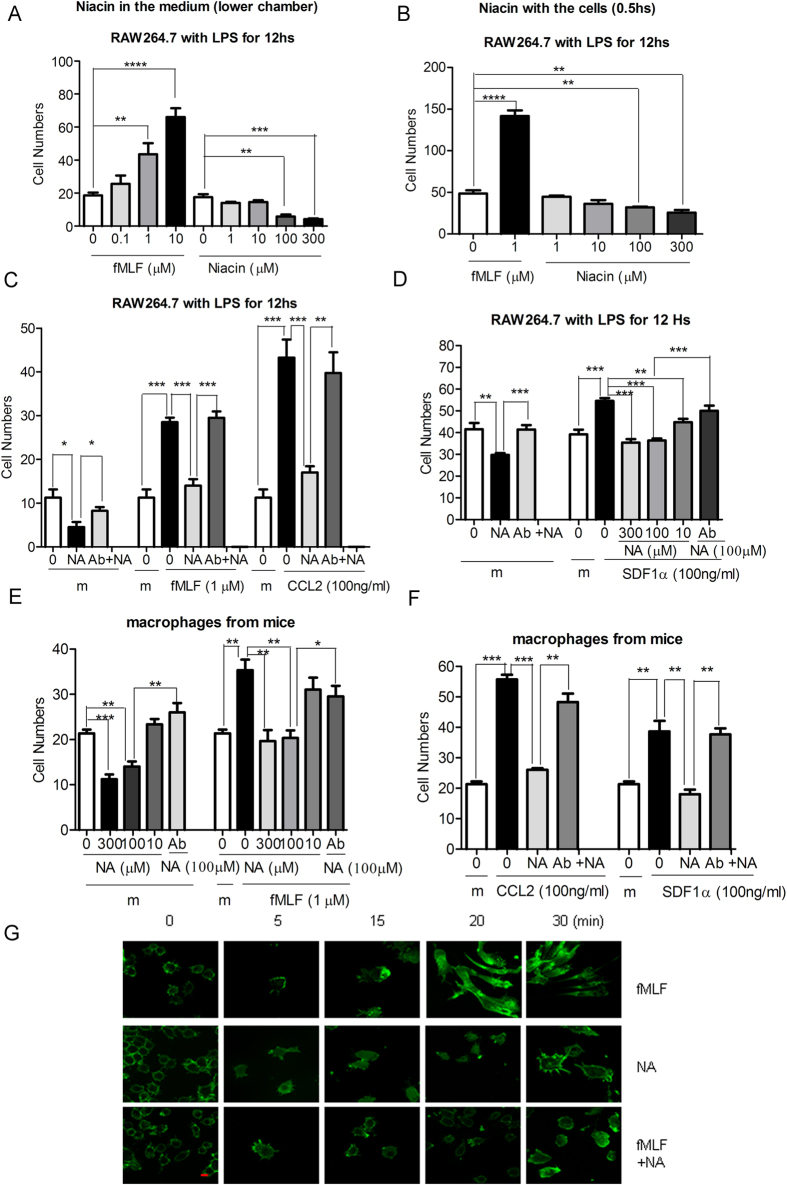
Niacin inhibited macrophage chemotaxis induced by fMLF, CCL2 and SDF1α (CXCL12). (**A–D**) RAW264.7 cells treated with LPS for 12 hs. (**E**,**F**) Primary macrophages isolated from mice. A. Niacin was placed in the lower wells of the chemotaxis chamber to test its chemotractic activity. (**B–F**) RAW264.7 cells were cultured with niacin for 30 min or pre-treated with HCA2 antibody for 30 min follewed by niacin. fMLF, SDF1α and CCL2 were then used to induce cell chemotaxis. The data are presented as the mean ± SE of triplicate values from an experiment with three to six repetitions (*p < 0.05; **p < 0.01; ***p < 0.001; ****p < 0.0001, compared to the indicated control). G. Zigmond chamber assay of LPS-treated RAW264.7 cells in response to fMLF gradient (0–10 μM), Niacin gradient (NA, 0–100 μM), and both fMLF and Niacin gradient (left groove: medium, right groove: 10 μM fMLF and 100 μM NA) for different times. F-actin was stained by FITC-phalloidin and images were acquired by confocal microscopy (bar: 10 μM).

Since niacin has been reported to possess anti-inflammatory activities, we next examined whether niacin may interfere with the chemotaxis of macrophages in response to bacteria and host-derived chemoattreactants, which is a hallmark of inflammation. Figure 2 shows that niacin did not induce the migration of macrophages with or without LPS treatment, while a bacterial chemotactic peptide fMLF induced significant migration of macrophages. In contrast, niacin present either in the upper wells (Fig. 2A) or lower wells of the chemotaxis chamber (Fig. 2B) caused a significant decrease in the chemotaxis of LPS-stimulated RAW264.7 cells in response to fMLF. Moreover, treatment of LPS-stimulated RAW264.7 cells with niacin for 30 min resulted in complete inhibition of chemokine- and fMLF-induced macrophage migration. The inhibitory effect of niacin on macrophage migration was reversed by pretreatment of the cells with an anti-HCA2 antibody suggesting the involvement of HCA2 in the biological activities of niacin on macrophages (Fig. 2C and D). The naicin-mediated inhibition of chemoatractant-induced migration was also observed in primary macrophages isolated from the peritoneal cavity of mice, and the effect of niacin was similarly reversed by the specific antibody against HCA2 (Fig. 2E and F).

Since chemoattractant receptor activation triggers actin cytoskeleton reorganization to form lamellipodia, we examined the distribution of F-actin in response to chemoattractant and niacin stimulation. Figure 2G shows that fMLF induced a markedly polarized subcellular F-actin localization at the leading edge of macrophages, whereas niacin induced polymerization of F-actin but failed to reorganize actin cytoskeleton. Rather, niacin inhibited fMLF-stimulated formation of F-actin-rich lamellipodia (pseudopodia) at the leading edge of the cells. Niacin did not affect the viability (Supplement data 1) and phagocytosis (Supplement data 2) of macrophages suggesting that it interfered with the signaling cascade of chemoattractants in macrophages.

**Figure 3:**
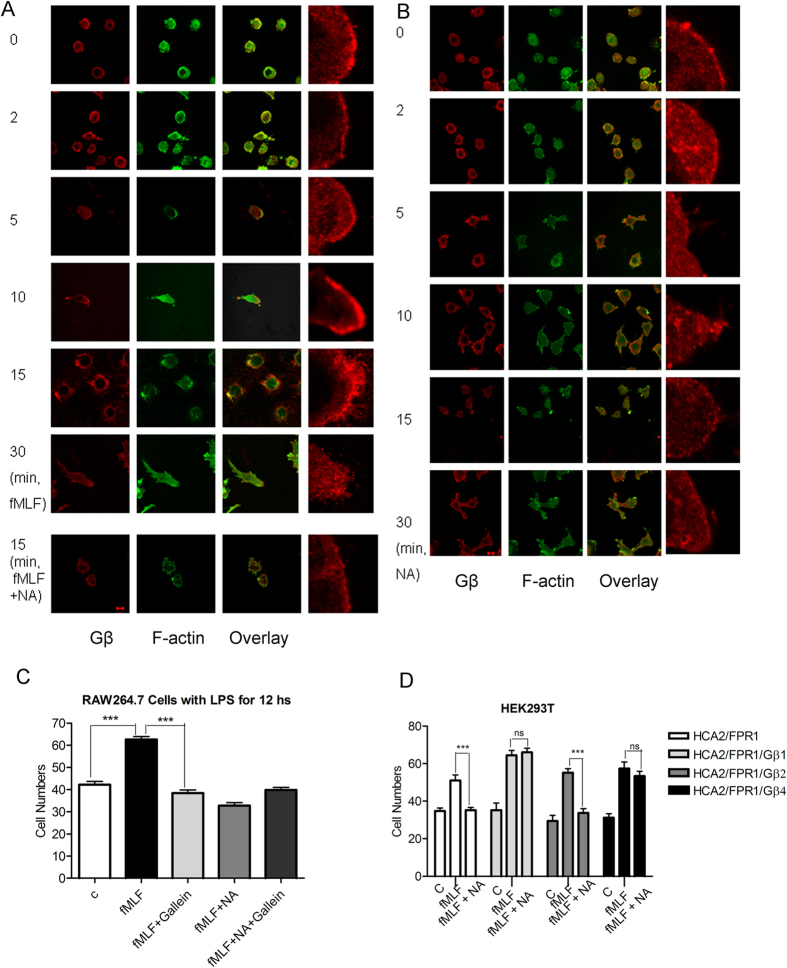
Gβγ is required for the inhibitory activity of niacin on fMLF-induced chemotaxis of RAW264.7 cells and FPR1 transfected HEK293T cells. The cellular localization of Gβ after stimulation by gradients of fMLF (**A**) or niacin (**B**) or both (A-bottom raw) on RAW264.7 cells using zigmond chamber assays in revealed by staining with rabbit anti-Gβ antibodies followed by an Alexa 568-conjugated anti-rabbit igG secondary antibody. F-actin was stained with FITC-labeled phalloidin (bar: 10 μM). (**C**) Cell chemotaxis was inhibited by the Gβγ inhibitor gallein. (**D**) Cell chemotaxis was not inhibited by niacin in HCA2/FPR1/Gβ1 and HCA2/FPR1/Gβ4 transfected HEK293T cells.

Gβγ subunits dissociated from pertussis toxin-sensitive G_i_ protein have been shown to play an essential role in the regulation of HCA2 internalization and ERK1/2 activation^35^,^36^. Previous studies showed that chemoattractant receptors were uniformly distributed on plasma membrane undergoing chemotaxis, but membrane-associated Gβ subunits of G proteins were localized in a shallow anterior-posterior gradient^37^.We then examined the cellular localization of Gβγ together with F-actin during cell chemotaxis. Confocal microscopy showed that LPS-treated RAW264.7 cells assumed a round shape, with Gβγ subunits (red) evenly distributing in the plasma membrane without chemoattractant stimulation. Following stimulation by fMLF (Fig. 3A), but not by niacin (Fig. 3B), the cells became well polarized, with the Gβγ subunits co-localized with F-actin-rich (green) pseudopodia at the leading edge of the cells. Niacin treatment disrupted fMLF-induced cell polarization and the polarized distribution of Gβγ (Fig. 3A). Taken together, our findings suggest that loss of polarized distribution of Gβγ is involved in the chemotaxis of macrophages and niacin-mediated inhibition. We also transfected Gβ1, Gβ2 or Gβ4 into HCA2/FPR1-expressing HEK293T cells to study chemotaxis in response to fMLF. Overexpression of Gβ1 and Gβ4 resulted in a significant reduction in niacin-induced inhibition of cell migration in response to fMLF. However, Gβ2 overexpression did not significantly change the inhibitory effect of niacin (Fig. 3C). The results were confirmed by a Gβγ inhibitor, gallein^17^ (Fig. 3D), which inhibited fMFL-induced cell chemotaxis regardless of the presence of niacin. Our results indicate that Gβγ is necessary for cell migration, consistent with previous reports^3^.

### ERK1/2 is involved in the inhibition of chemoattractant-induced migration of macrophages by niacin

**Figure 4:**
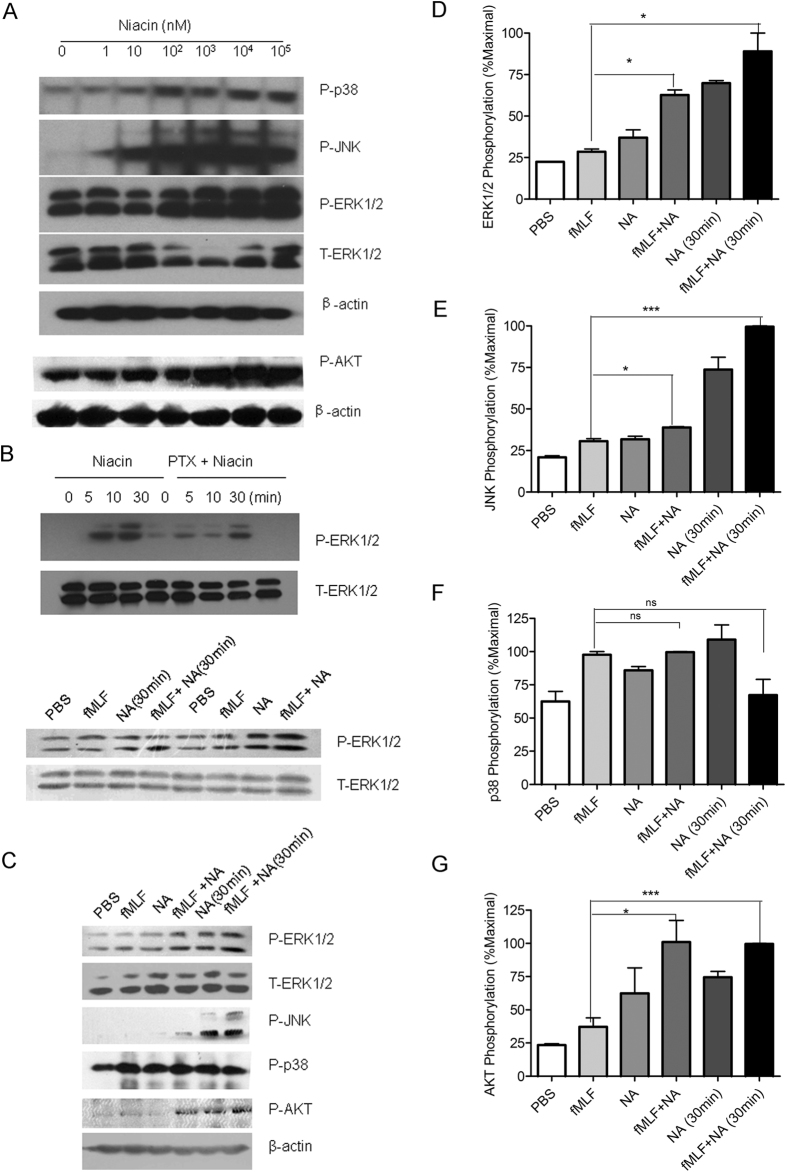
ERK1/2, JNK, P38, and AKT activation induced by fMLF and niacin in RAW264.7 cells treated with LPS for 12 hs. (**A**) Cells treated with different concentrations of niacin for 5 min. (**B**) Cells treated with 100 μM niacin for different times. Cells pre-treated with PTX (50 ng/ml) overnight were used as the control. (**C–G**) Additive effects of ERK1/2, JNK and AKT phosphorylation induced by fMLF and NA. RAW264.7 cells were treated with PBS (5 min, lane 1), 1 μM fMLF (5 min, lane 2), 100 μM NA (niacin, 5 min, lane3), fMLF and NA (5 min, lane 4), or 100 μM NA (30 min, lane5), fMLF and NA (5 min; 30 min, lane 6). ERK1/2, JNK, p38 and AKT phosphorylation was detected by Western blot (**C**). ERK1/2, JNK, p38 and AKT phosphorylation was quantified by densitometric analysis and normalized against loading control (T-ERK or β-actin) (**D–G**). The phosphorylation is expressed as the percentage of the maximal signal in the same experiment. Statistical differences were analyzed with t-test (PRISM software) (*p < 0.05; **p < 0.01, ***p < 0.001, ns: no significant difference, between the two indicated groups).

GPCR-mediated activation of mitogen-activated protein kinases (MAPKs) and PI3K/Akt is involved in the regulation of cell migration^38^,^39^. In our study, we observed that niacin induced the activation of p38 (T180/Y182), JNK (Thr183/Tyr185), ERK1/2 (42/44) and AKT (T308) in RAW264.7 macrophages (Fig. 4A). The phosphorylation of ERK1/2 induced by niacin was inhibited by pertussis toxin (PTX) indicating the involvement of Gi-type G-proteins in the effect of niacin (Fig. 4B). To investigate the potential interaction of signaling pathways induced by fMLF and niacin, we pre-cultured LPS-treated RAW264.7 cells with niacin for 30 min, followed by addition of fMLF for 5 min, or co-stimulated the cells with niacin and fMLF for 5 min, then examined the phosphorylations of ERK1/2, JNK, p38 and AKT. As shown in Fig. 4, fMLF-induced phosphorylation of ERK1/2, JNK and AKT was increased when the cells were co-incubated with niacin for 5 min or pre-treated for 30 min, while p38 phosphorylation was unchanged (Fig. 4C–G). Thus, niacin shows a selected additive effect on chemoattractant-induced activation of ERK1/2, JNK and PI3K pathways.

**Figure 5:**
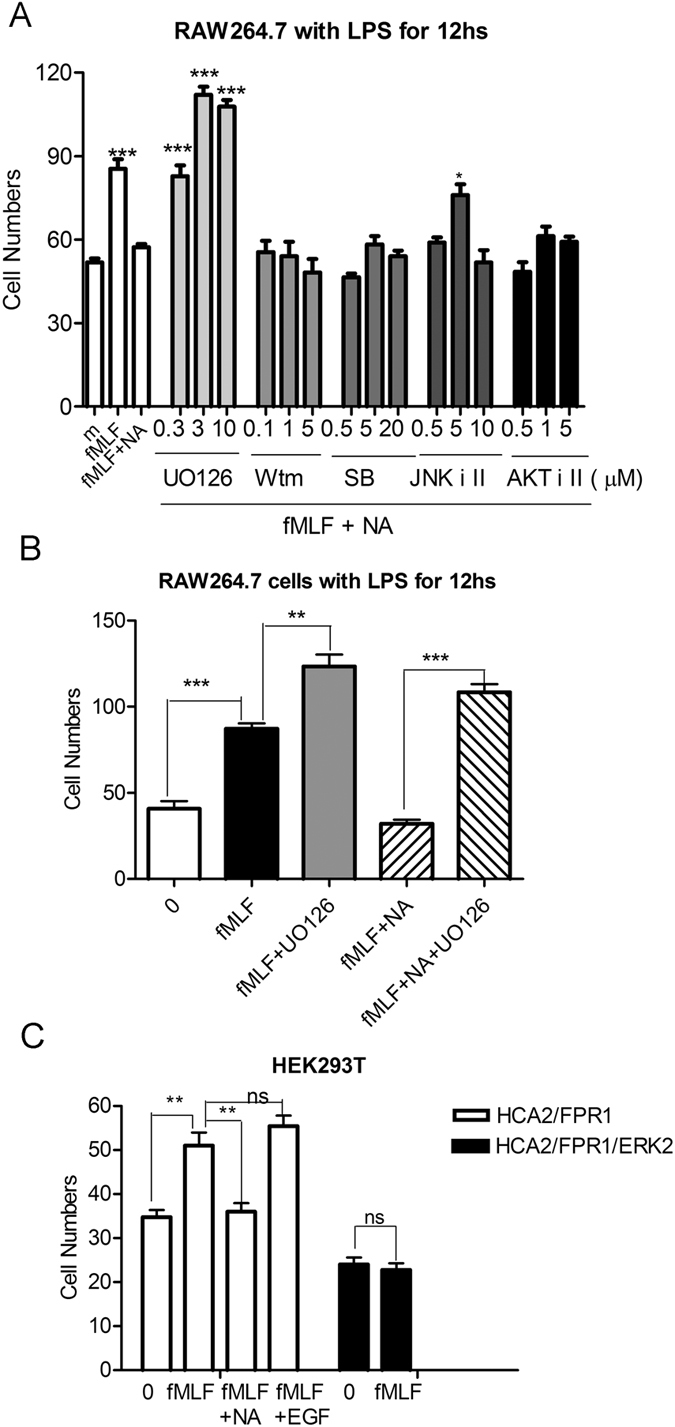
Chemotaxis of macrophages in the presence of niacin and kinase inhibitors. (**A**) RAW264.7 cells treated with LPS for 12 hs were pre-incubated with the ERK1/2 inhibitor UO126, the PI3K inhibitor Worttmannin, the p38 inhibitor SB203580, JNK inhibitor II and AKT inhibitor II for 15 min. Then the cells were cultured with 100 μM niacin for another 30 min. fMLF was used as a chemoattractant in all experiments. *p < 0.05; **p < 0.01, ***p < 0.001, compared with the cells cultured with 100 μM niacin and fMLF. (**B**) UO126 enhanced cell chemotaxis induced by fMLF. (**C**) EFG failed to inhibit the chemotaxis of HCA2/FPR1 co-transfected HEK293T cells. fMLF failed to induce chemotaxis of HCA2/FPR1/ERK2 co-transfected HEK293T cells.

To explore the role of ERK1/2, JNK and AKT induced by niacin in the regulation of macrophage migration, LPS-treated RAW264.7 cells were pre-incubated with the ERK1/2 inhibitor UO126, the PI3K inhibitor Worttmannin, the p38 inhibitor SB203580, the JNK inhibitor II and the AKT inhibitor II, respectively, followed by measurement of cell chemotaxis in the presence of niacin. The MEK inhibitor UO126 not only increased fMLF-induced macrophage chemotaxis, but also reduced niacin-mediated inhibition of macrophage chemotaxis in response to chemoattractants. However, JNK, AKT and p38 inhibitors did not show such effects on niacin-mediated inhibition of macrophage migration induced by chemoattractants (Fig. 5A and B). In an attempt to clarify the mechanisms of niacin-mediated inhibition of macrophage chemotaxis, we found that overexpression of ERK2 led to a significant decrease in fMLF-induced chemotaxis of HEK293T cells co-transfected with HCA2 and the high affinity fMLF receptor FPR1 (Fig. 5C), suggesting that as a negative regulator, niacin-induced activation of the ERK1/2 signaling pathway may contribute to the inhibition of fMLF-induced chemotaxis of RAW264.7 cells and of HEK293T cells expressing FPR1.

Our previous studies demonstrated that stimulation of HCA2 by niacin resulted in the dissociation of G_i_ proteins from G_βγ_-subunits, causing the PKC pathway to couple to ERK1/2 ( ≤ 2 min) and MMP/EGFR transactivation at later time points (2–5 min). We therefore tested the role of EGFR transactivation-depenedent ERK1/2 signaling in the regulation of macrophage migration. As shown in Fig. 5C, EGF alone did not inhibit fMLF-mediated migration of HCA2/FPR1 co-expressing HEK293T cells. Thus, it is likely that niacin-triggered PKC-dependent ERK1/2 activation, but not EGFR transactivation-dependent ERK1/2 pathway, was involved in the inhibition of chemoattractant-induced migration of macrophages by niacin.

### Niacin induces heterologous desensitization and internalization of FPR1

**Figure 6:**
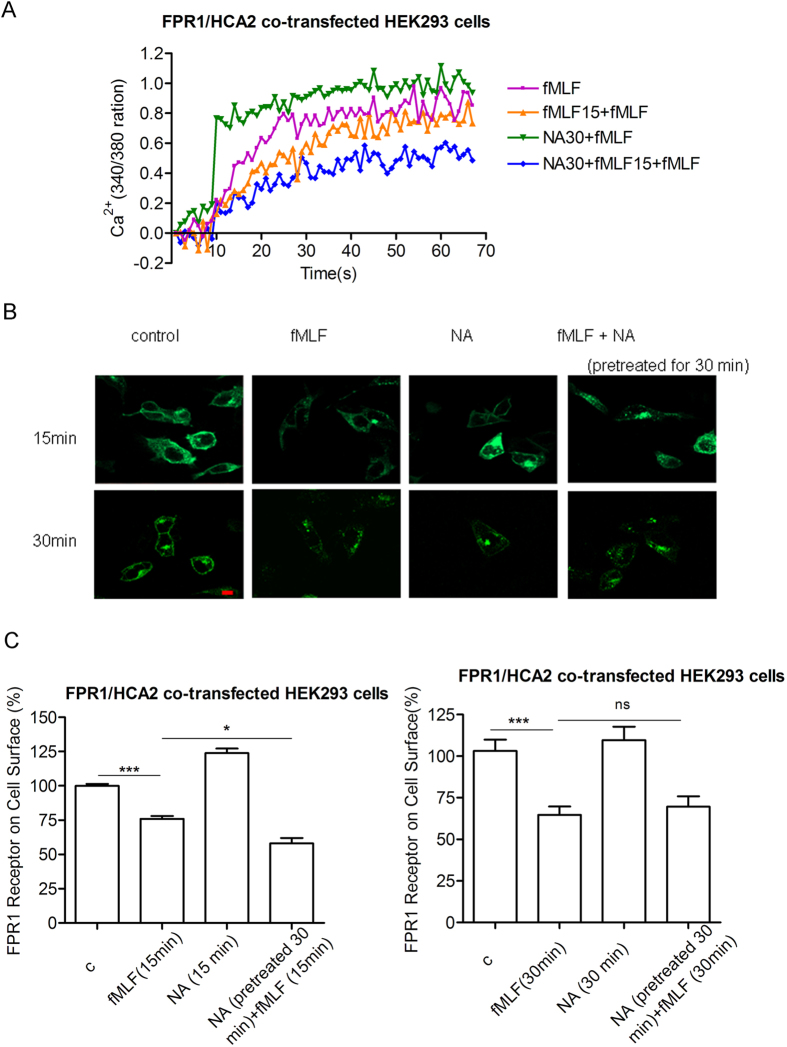
Niacin induces internalization and heterologous desensitization of FPR1. (**A**) HEK293 cells co-expressing FPR1-EGFP and flag-HCA2 were loaded with the calcium probe Fura-2/AM, followed by the first or second stimulation (15 min later) of 5 μm fMLF with or without pretreated with 300 μm niacin, then calcium mobilization was assayed by monitoring the change in Fura-2/AM fluorescence. All data shown are representative of at least four independent experiments. (**B**) HEK293 cells co-expressing FPR1-EGFP and flag-HCA2 were stimulated with 5 μm fMLF with or without 300 μm niacin for 15 min or 30 min, then internalization of FPR1-EGFP was observed by confocal. (**C**) ELISA measurement of FPR1 remaining on the cell surface after treatment of cells by 5 μm fMLF with or without 300 μm niacin for 15 min or 30 min. Error bars, S.E. for four replicates. Data were analyzed using Student’s t test (**p < 0.01;***p < 0.001).

It has been reported that pretreatment with adenosine compounds desensitized chemokine RANTES-induced chemotaxis and Ca^2 + ^flux through activation of A2a adenosine receptor^40^. We therefore investigated whether HCA2 also induced heterologous desensitization of chemoattractant receptors. As shown in Fig. 6A, when HEK293 cells co-expressing FPR1 and HCA2 were pre-incubated with niacin for 30 min, fMLF stimulation led to slight increase in Ca^2 + ^mobilization compared with the cells in the absence of niacin. But when the cells were pre-treated with fMLF and niacin, the second fMLF stimulation resulted in a more significant decrease in Ca^2 + ^mobilization in the cells with niacin than the cells without niacin. Further investigation revealed that niacin alone did not induce FPR1 internalization, but when the cells were pretreated with niacin, fMLF caused more rapid FPR1 internalization (Fig. 6B). As shown in Fig. 6C, fMLF treatment for 15 min induced about 25% FPR1 to internalize from cell surface into the intracellular compartment without niacin, whereas almost 40% FPR1 internalized in response to fMLF in the presence of niacin. Our results thus suggest that activation of HCA2 induces more rapid internalization of FPR1 and heterologously desensitizes fMLF-induced Ca^2 + ^mobilization.

### Niacin suppresses LPS-induced IL-6 secretion and up-regulates PDG2 production by macrophages

**Figure 7:**
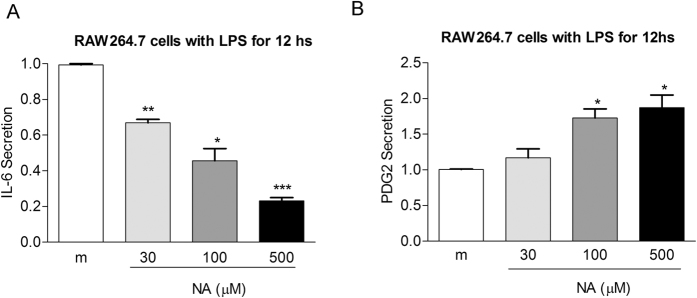
The effect of niacin on IL-6 (**A**) and PDG2 (**B**) release by RAW264.7 cells treated with LPS. RAW264.7 cells stimulated by LPS were exposed to niacin at different concentrations overnight. The medium was collected for IL-6 and PDG2 measurement. Results shown are the mean ± SD of quadruplicate measurements from one representative experiment out of three performed (*p < 0.05; **p < 0.01, ***p < 0.001, compared with the control groups).

To determine the effect of niacin on proinflammatory cytokine production by macrophages, RAW264.7 cells were exposed to LPS with or without the addition of niacin. Exposure of the cells to LPS resulted in significant release of IL-6 and TNF-α into the supernatant. Addition of niacin in the culture attenuated the ability of macrophages to produce IL-6 in response to LPS (Fig. 7A), without an effect on TNF-α production (data not shown). Niacin also induced PGD2 release from LPS-treated RAW264.7 (Fig. 7B). These results suggest that niacin selectively inhibits inflammatory responses of macrophages triggered by LPS. It appears that IL-6 and IL-1β may induce the expression of HCA2 in macrophages, which in turn inhibits the secretion of inflammatory cytokines such as IL-6.

### Niacin protects mice from acute septicemia

**Figure 8:**
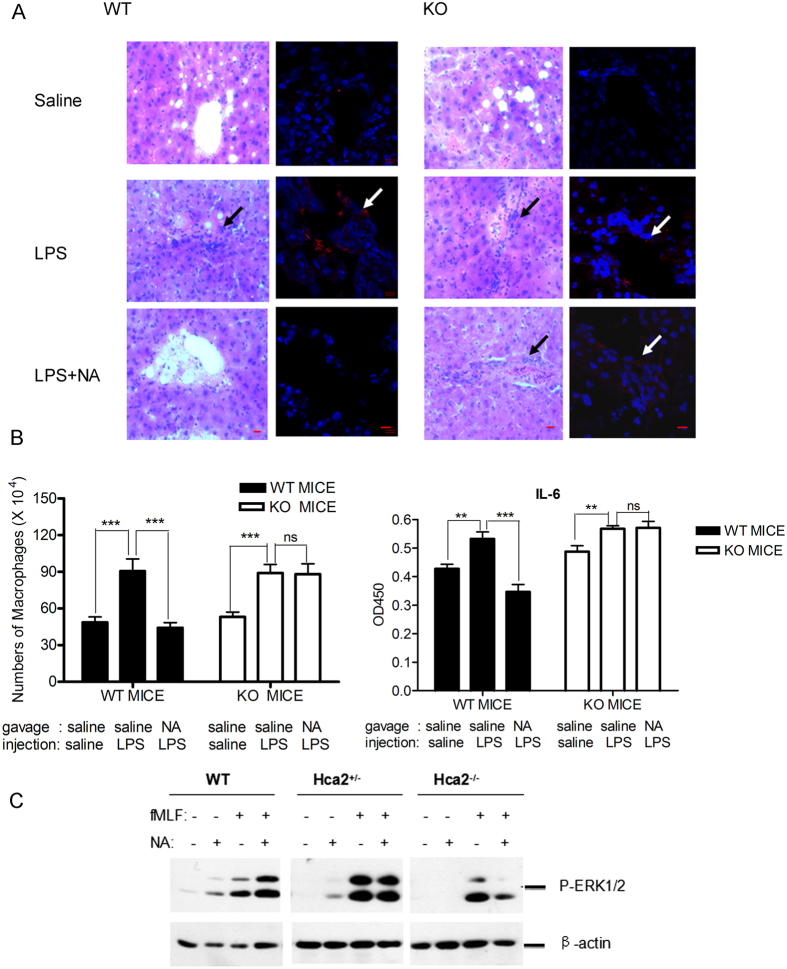
Niacin inhibits LPS-induced acute endotoxemia in mice. Hca2 KO and WT mice were sacrificed 4 h following endotoxemia induction. (**A**) HE staining (original magnification, 400×, bar: 10 μM) and immunofluorescent staining by anti-F4/80 antibody (original magnification, 630×, bar: 10 μM) of liver sections from saline-, LPS- or LPS + niacin-treated WT and Hca2^−/−^ mice. (**B**) The number of macrophages isolated from the ascites of WT and Hca2^−/−^ mice (n = 10) and IL-6 in the ascites of WT and Hca2^−/−^ mice treated with saline, LPS, or LPS + niacin (n = 8–10). Error bars represent standard deviation of the means (*p < 0.05; **p < 0.01, ***p < 0.001, compared with the indicated group). (**C**) ERK1/2 activation in niacin-treated macrophages from WT, Hca2^+/−^ and Hca2^−/−^ mice.

Based on the ability of niacin to inhibit the proinflammatory responses of macrophages, we examined whether niacin interfered with the progression of LPS-induced endotoxemia *in vivo*. As shown in Fig. 8, in wild-type mice and Hca2^−/−^ mice, LPS injection caused inflammatory symptoms with significant increases in the number of macrophages in the peritoneal cavity and IL-6 secretion in ascites. Mouse liver sections showed significant infiltration of inflammatory cells. Niacin pretreatment markedly diminished the number of macrophages and IL-6 level in the ascites as well as inflammatory cell infiltration in the liver. In contrast, niacin failed to show any effect on LPS-induced endotoxemia in Hca2^−/−^ mice (Fig. 8A and B). Also, niacin did not activate ERK1/2 or enhanced fMLF-induced ERK1/2 phosphorylation in macrophages isolated from Hca2^−/−^ mice (Fig. 8C). These results indicate the ability of niacin to inhibit the development of LPS-induced acute endotoxemia through HCA2.

## Discussion

Niacin has been known to exert favorable effects on the levels of plasma lipoprotein, by reducing atherogenic lipoproteins LDL-c, VLDL-c, and Lp(a) but raising HDL-c^1^,^3^. In humanized mice lacking the LDL receptor, niacin administration did not result in the reduction of plasma free fatty acids, but still exhibited effects on HDL-c, LDL-c and triglycerides, suggesting a HCA2-independent mechanism for niacin to modify dyslipidemia^17^. In addition, HCA2 has been found to be expressed by various immune cells including macrophages, neutrophils, and epidermal Langerhans cells^19^,^20^,^41^,^42^, and the expression in macrophages is increased by treatment with IFN-γ in combination with TNF-α, lipopolysaccharide, and CpG oligodeoxynucleotides^20^. Recent studies revealed that niacin possesses anti-inflammatory activities in various tissues/cell types via HCA2^18^,^27^,^28^,^29^,^30^,^43^,^44^. Interestingly, activation of HCA2 by niacin significantly inhibited chemokine-induced migration of macrophages^18^,^34^. Macrophages and their precursors, monocytes, are critical effectors of the innate immune system that protect the host by infiltrating inflammatory sites and killing pathogenic microbes. However, macrophages also orchestrate harmful processes associated with inflammatory disorders^45^,^46^. Macrophage migration in response to chemotactic ligands is a key event in inflammation, and is controlled by chemoattractant GPCRs^47^. Although niacin and chemoattractants exert their biological activities through similar receptors coupled to Gi-type G-proteins^48^,^49^, niacin is not chemotactic but instead inhibits chemoattractant-mediated chemotaxis of macrophages. These divergent activities prompted us to clarify the signaling cascade downstream of HCA2 involved in the inhibition of chemoattractant GPCRs in macrophages.

It is well eatablished that upon activation by cognate ligands, Gi-coupled GPCRs exert their activity through the inhibition of adenylyl cyclase, and regulate distinct downstream effector molecules, including certain isoforms of adenylyl cyclase, phospholipase C-β (PLC-β), phosphatidylinositol 3-kinase (PI3K), p21- activated kinase, and GPCR kinases, via Gβγ subunits uncoupled from PTX-sensitive Gi proteins^50^,^51^,^52^. This was shown by experiments with Dictyostelium in which the Gβ-null mutants were severely defective in development, chemotaxis, and phagocytosis. The observation that blockade of Gβγ signaling resulted in tumor growth in an experimental lulng tumor metastasis model indicates that Gβγ subunits are also required in later steps of tumor metastatic cascade that involves tumor cell motility^53^. Recent studies showed that the selectivity of the Gβγ dimer on its interaction with effectors such as PLC-β^54^, as well as in the regulation of neutrophil function^55^ depends on the Gβ identity. Gβγ subunits were found to be partially immobilized and confined in a F-actin-dependent fashion^56^. Our previous studies revealed that Gβγ dissociated from PTX-sensitive Gi plays an essential role in the recruitment of GRK2 to phosphorylate the C-terminus of HCA2 and subsequent ERK1/2 activation^37^,^38^. In the present study, overexpression of Gβ1 and Gβ4 attenuated the inhibitory effect of niacin on fMLF-mediated migration of HEK293 cells over-expressing FPR1. Treatment with gallein, a small molecule inhibitor of Gβγ function, deceased the inhibitory effect of niacin on chemoattractant-mediated cell migration. These results suggest a key role of Gβγ in the regulation of cell chemotaxis in response to GPCR ligands. On the one hand, it is possible for Gβγ to regulate cell chemotaxis through GRK2-mediated GPCR desensitization, and/or activation of ERK1/2. It is also possible that HCA2 competes with chemoattractant receptors for the same Gβγ to relay signals. Further studies are required to more clearly delineate the role of Gβγ in regulating GPCR-mediated cell chemotaxis.

MAPKs are a family of serine/threonine kinases, including ERK1/2, p38 MAPK, and JNK^57^. Activation of MAPKs is one of the key components in signal transduction associated with cell chemotaxis^58^,^59^. Previous studies showed that activation of ERK1/2 and p38 MAPK contributes mainly to the chemotaxis induced by C5a in RAW264.7 macrophages^60^. Recent studies demonstrate that the phosphorylation of ERK1/2 potentiates the activity of GRK2, leading to inhibition of leukocyte migration, while MAPK p38 acts as a noncanonical GRK to facilitate cell migration by blocking GRK2 function^61^,^62^. Our present study clearly showed that niacin enhanced ERK1/2 activity in macrophages in the presence of chemoattractants, and pretreatment of the cells with the MEK1/2 inhibitor U0126 reduced the inhibitory effect of niacin on chemoattractant-induced cell migration. Our data also indicates that upon binding by niacin, HCA2 initially activates ERK1/2 via two distinct pathways, one is PKC-dependent, occurring at a peak time of ≤ 2 min, the other is MMP-dependent EGFR transactivation, occurring at both earlier and later time points (2–5 min)^38^. On examination of whether growth factor receptor transactivation-mediated ERK1/2 activation is involved in inhibiting chemoattractant-induced macrophage migration by niacin, we found EGF alone activates of ERK1/2, but without inhibitory effect on chemoattractant-induced chemotaxis of macrophages, suggesting that EGFR transactivation-induced ERK1/2 phosphorylation is not involved. Therefore, it is likely that HCA2 inhibits chemoattractant-induced macrophage migration via PKC-dependent ERK1/2 signaling cascade.

It is well established that stimulation of one GPCR by an agonist leads to inhibition of a nontargeted receptor signaling by a mechanism known as heterologous receptor desensitization^63^. Activation of μ- and δ-opioid receptors with agonists is able to heterologously desensitize chemokine receptors CCR1, 2 and 5, but not CXCR4^64^,^65^. Our results showed that co-stimulation with niacin reduced fMLF-mediated Ca^2 + ^mobilization, accompanied by significant internalization of FPR1. This is consistent with the observation showing that FPR1-mediated heterologous desensitization of CXCR4 and C5a receptors was accompanied by receptor internalization^66^,^67^. Previous studies established that the ligands of CXCR3^68^, CCR5^69^ and k-opioid receptor^64^ desensitize the chemotaxis of Th1 cells toward CXCL12. Our findings suggest that niacin-induced heterologous desensitization of FPR1 contributes to HCA2-mediated inhibition of macrophage migration. In recent years, protein kinase C (PKC)^70^, protein kinase A (PKA)^40^ and GRKs^71^ have been shown to be involved in heterologous GPCR desensitization. However, additional studies are needed to elucidate the signaling pathways involved in niacin-induced heterologous desensitization of FPR1.

Monocytes and macrophages are key mediators of inflammation, and their migration in response to chemotactic agonists is a crucial determinant of the inflammation processes. In agreement with the *in vitro* observations in LPS-treated RAW264.7 cells, we found that treatment of wildtype mice with niacin decreased the number of macrophages in LPS-elicited ascites, IL-6 secretion and the infiltration of macrophages into the liver, with no effect on Hca2-deficient mice. These findings indicate that niacin inhibits macrophage proinflammatory responses through HCA2. Since inflammation is considered a major cause of various diseases, such as atherosclerosis, diabetes, obesity, sepsis, and cancer, HCA2 and its signaling cascades may constitute valuable therapeutic targets.

## Methods

### Ethics Statement

All animal work was conducted in accordance with the Guide for the Care and Use of Laboratory Animals (United States National Institutes of Health). The protocol was approved by the research ethics committee of Zhejiang University (ZJU2010-1-01-020).

### Reagents

Niacin, LPS, IFN-γ and signaling molecule inhibitors were obtained from Sigma-Aldrich (St. Louis, MO). RPMI 1640 medium and fetal bovine serum (FBS) were purchased from Hyclone (Beijing, China). Lipofectamine 2000 and G418 were obtained from Invitrogen (Carlsbad, CA). pEGFP-N1 and pCMV-Flag vectors were purchased from Clontech Laboratories, Inc. (Palo Alto, CA) and Sigma, respectively. Primary antibodies for p-ERK1/2, p-p38, p-JNK, p-AKT, β-actin and total ERK were purchased from Cell Signaling (Danvers, MA).

### Cell line and mouse primary macrophage culture

RAW264.7 macrophages were cultured in RPMI 1640 medium supplemented with 10% FBS, 100 U/mL penicillin, and 100 μg/mL streptomycin in a humidified atmosphere of 95% air and 5% CO2 at 37 °C. To obtain primary macrophage, wild-type (WT) and Hca2-deficient C57BL/6 mice were intraperitoneally injected with 1.5 mL of 3% Brewer thioglycollate (Sigma). Peritoneal macrophages were harvested by using cold PBS 72 hs later. The harvested cells were washed with PBS and cultured in RPMI 1640 medium supplemented with 10% FBS at 37 °C overnight to collect adherent cells for further assays.

### RT-PCR

Total RNA was isolated from cultured and primary macrophages with TRIzol reagent (Invitrogen, Carlsbad, CA). RT-PCR was conducted to detect the expression of Hca2 by using primers 5′-GGC GTG GTG CAG TGA GCA GT-3′ (forward), and 5′-GGC CCA CGG ACA GGC TAG GT (reverse).

### Western blot

Cells were grown in 6-well plates and were serum starved for 2 hs in culture medium prior to stimulation with indicated stimulants at designated concentrations. The cells were subsequently lysed in 100 μl lysis buffer [20 mM HEPES (pH 7.5), 10 mM EDTA, 150 mM NaCl, 1% Triton X-100], with protease inhibitors (Roche, Indianapolis, IN) at 4 °C on a rocker for 30 min. The lysates were separated by 12% SDS-PAGE and blotted onto polyvinylidene difluoride membranes, which were blocked for 1 h at room temperature in TBST (10 mmol/L Tris, 150 mmol/L NaCl, 0.1% Tween-20, pH 8.0) buffer containing 5% (v/w) skim milk. Membranes were then probed overnight with primary antibodies in TBST containing 5% (v/w) BSA, and then incubated with a horseradish peroxidase-labeled secondary antibody (1:5,000) for 1 h at room temperature in TBST containing 5% (v/w) milk powder. Immunoreactivity was detected by ECL.

### Chemotaxis assay

Macrophages were washed twice in migration buffer (RPMI 1640 with 0.1% fatty acid-free BSA and 10 mM HEPES, pH7.4) and resuspended at 2 × 10^6^ cells/ml in the migration buffer. The cells were then treated with inhibitors or niacin for the indicated time at 37 °C. 48-well chemotaxis chambers with 8-μm pore size polycarbonate filters (Neuro Probe, Gaithersburg, MD) were used for the assays. Chemoattractants were diluted and added into the lower wells of the chambers. Cells were added into the upper wells of the chambers. The upper and lower chambers were separated with polycarbonate filters. After 4 hs at 37 °C in 5% CO2, the cells remaining on the upper surface of the filters were removed with a cotton swab. Migrated cells attaching to the lower surface of the filters were fixed with 75% ethanol for 30 min, and stained with Diff-Quik. The number of migrated cells was counted under microscope. For each sample, cells in 6 randomly picked fields under 400 x magnification were counted.

### Zigmond chamber assay

Zigmond chambers were purchased from Neuro Probe and the assays were performed following the manufacturer’s instructions. Briefly, macrophages were cultured on a coverslip, which was mounted onto a grooved microscope slide with cell-covered portion centered over the bridge. Chemoattractants or control migration buffer was added to fill the grooves. The chamber was then incubated at 37 °C for 15 or 30 min. The cells were fixed with 4% paraformaldehyde for 10 min, followed by F-actin staining with FITC-Phalloidin for 30 min. Cell orientation and morphological images were acquired using confocal microscopy (Zeiss LSM 510) with an attached Axiovert 200 microscope and a LSM5 computer system.

### Histopathology and Immunofluorescent Staining

Mouse liver tissues were immediately embedded with O.C.T compound and analyzed using frozen-sections (6 μm). For histopathology, the sections were stained by hematoxylin and eosin (HE). For immunofluorescence, the sections were fixed and permeabilized with cold acetone and 0.1% Triton-x-100, then blocked with 5% goat serum in 0.01 M PBS containing 0.3% Triton X-100 for 2 hs at RT. The sections were incubated overnight at 4 °C with a rabbit anti-F4/80 antibody (Santa Cruz) followed by an Alexa Fluor 555-conjugated goat anti-rabbit IgG (1:500; Beyotime) for 2 hs at RT. Nucleus was stained with 0.01 M PBS containing 10 μg/ml 4′,6-diamidino-2-phenylindole (DAPI). Images were taken with a Zeiss LSM 510 microscope.

### IL-6 and PDG2 secretion

IL-6 and PGD2 in the culture medium of macrophages were assayed by ELISA (CUSABIO, Wuhan, China) according to the manufacturer’s instructions.

### LPS-induced acute endotoxemia in mice

Animals were housed in the Laboratory Animal Center of Zhejiang University. Hca2^−/−^ mice were obtained by intercrossing Hca2^+/−^ mice. Genotyping of the Hca2 alleles was performed by PCR using the primers Hca2-sense-1(5′-TCA GAT CTG ACT CGT CCA CC-3′) and 333-KO (5′-CCT CTT CGC TAT TAC GCC AGC-3′) for the inactivated allele and the primers Hca2-sense-1 and 333-WT (5′-CCA TTG CCC AGG AGT CCG AAC-3′) for the wild-type allele as previous reported^12^.

For entoxemia, 4–6 week-old Hca2 KO (C57BL) and WT (C57BL) mice were used. Mice were divided into three groups (n = 8–12 each group): saline gavage with saline injection, saline gavage with LPS (10 mg/kg) injection and niacin (200 mg/kg) gavage with LPS injection. Mice were sacrificed 4 h following endotoxemia induction.

### Statistical analysis

All experiments were performed at least 3 times with consistent results. Results shown are from representative experiments. Statistical analysis was performed using GraphPad Prism VERSION 5 for Windows (GraphPad Software). Differences among the means (±SE) were evaluated by t-test. P < 0.05 was considered statistically significant.

## Additional Information

**How to cite this article**: Shi, Y. *et al*. Activated niacin receptor HCA2 inhibits chemoattractant-mediated macrophage migration via Gβγ/PKC/ERK1/2 pathway and heterologous receptor desensitization. *Sci. Rep.* **7**, 42279; doi: 10.1038/srep42279 (2017).

**Publisher's note:** Springer Nature remains neutral with regard to jurisdictional claims in published maps and institutional affiliations.

## Supplementary Material

Supplementary Dataset 1 and 2
