# Supplementary material for: Activated niacin receptor HCA2 inhibits chemoattractant-mediated macrophage migration via Gβγ/PKC/ERK1/2 pathway and heterologous receptor desensitization
Source: Sci Rep. 2017 Feb 10;7:42279. doi: 10.1038/srep42279 (PMC5301212; doi:10.1038/srep42279)
Supplement: Supplementary Dataset 1 and 2 [file srep42279-s1.doc]

**Activated niacin receptor HCA2 inhibits chemoattractant**

**-mediated macrophage migration via Gβγ/PKC/ERK1/2**

**pathway and heterologous receptor desensitization**

Ying Shi^1^, Xiangru Lai ^1^, Lingyan Ye^1^, Keqiang Chen^2^, Zheng Cao^1^, Wanghua Gong^2^, Lili Jin^1^, Chunyan Wang^2,3^, Mingyong Liu^2,4^, Yuan Liao^1^, Ji Ming Wang^2^***, Naiming Zhou^1^***

**1** *College of Life Sciences, Zhejiang University, Yu Hang Tang Load 388, Hangzhou, PR China;*

**2***Cancer and Inflammation Program, Center for Cancer Research, National Cancer Institute-Frederick, NIH, Frederick, MD 21702, USA;*

**3** *Xuzhou Yes Biotech Laboratories Ltd. Xuzhou, Jiangsu, PR China;*

**4** *Department of Spine Surgery, Daping Hospital, Third Military Medical University, Chongqing, PR China;*

***Corresponding Authors:

Dr. Naiming Zhou: College of Life Sciences, Zhejiang University, Zijingang Campus, 388 Yuhang Tang Road, Hangzhou, 310058, China. Phone: 0571-88206748; Fax: 0571-88206134-8000; E-mail: [zhounaiming@zju.edu.cn](mailto:zhounaiming@zju.edu.cn).

Dr. Ji Ming Wang: Cancer and Inflammation Program, Center of Cancer Research, National Cancer Institute-Frederick, NIH, Frederick, MD 21702. Phone: 301-846-6979; Fax: 301-846-7402; E-mail: [wangji@mail.nih.gov](mailto:wangji@mail.nih.gov);

Supplemental data 1


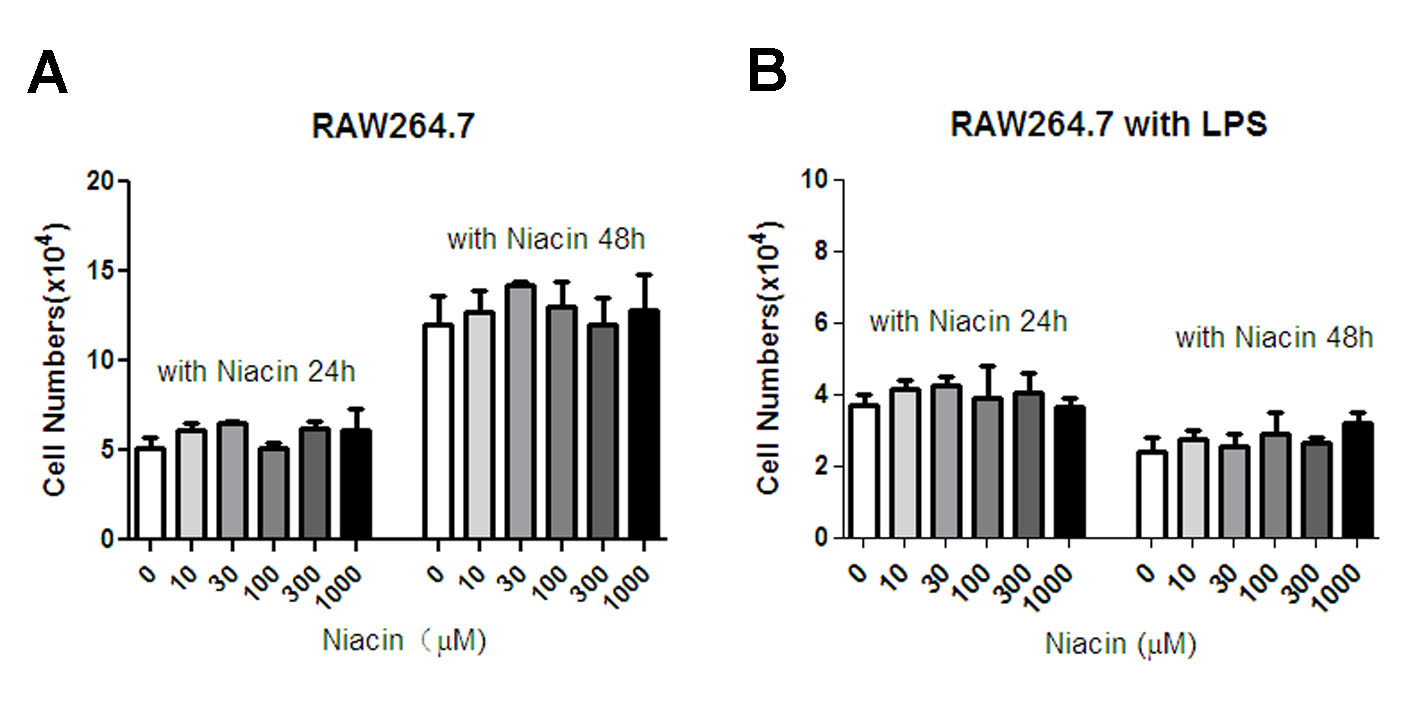


The influence of niacin on cell viability. Naive RAW264.7 cells and LPS-treated RAW264.7 cells were incubated with niacin at different concentrations for 24 h and 48 h. Live cells were then counted after staining by trypan blue under a microscope.

Supplemental data 2


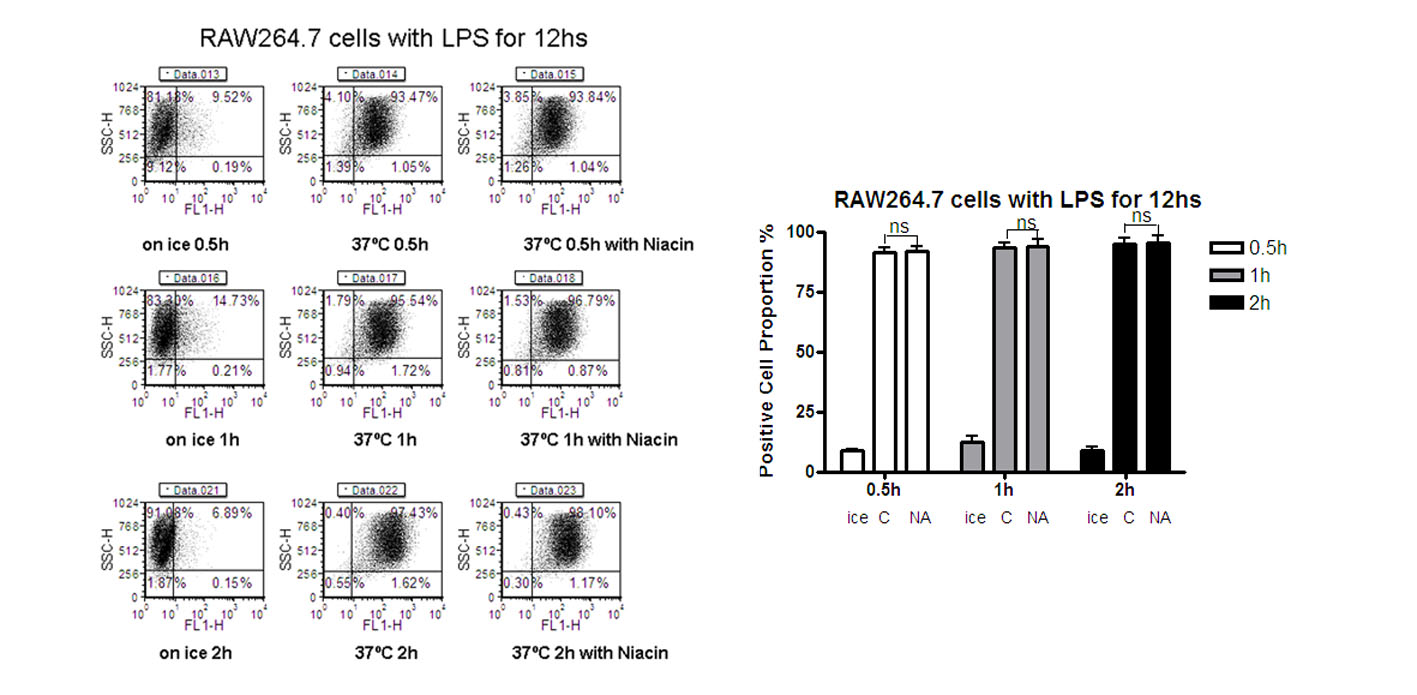


The influence of niacin on FITC-labeled dextran phagoecytosis by LPS-treated RAW264.7 cells. LPS-treated RAW264.7 cells were incubated with FITC-labeled dextran for 0.5, 1 and 2 h at 37ºC with or without niacin (100 μM). The cells were then analyzed for % positivity by flowcytometry. Cells with FITC-labeled dextran on ice were used as control.
